# Supplementary material for: Content-rich biological network constructed by mining PubMed abstracts
Source: BMC Bioinformatics. 2004 Oct 8;5:147. doi: 10.1186/1471-2105-5-147 (PMC528731; doi:10.1186/1471-2105-5-147)
Supplement: Additional File 2 — The original results of the above study (non-essential files are deleted to keep the file size under the limit set by BMC bioinformatics). [file 1471-2105-5-147-S2.bz2 › chilibotAdditionalFile2/dip30/355ID8120391E4997/html/IL10_IL10RA.html]

 


 **IL10** and **IL10RA** 
  
Found 91 abstracts in PubMed, retrieved 30.  
 

 What does Google say? 
 PDF only 
| .edu only 

---

**Interactive relationship** (e.g. stimulation, inhibition, etc)

**Neutral relationship**- Crystal structure of human cytomegalovirus IL 10  [ **IL10** ]  bound to soluble human IL 10R1  [ **IL10RA** ] .  Ref: 12093920 Proc Natl Acad Sci U S A, 2002
- IL 10  [ **IL10** ]  knockout and normal mice treated prophylactically with anti IL 10R  [ **IL10RA** ]  demonstrated accelerated granuloma assembly and rapid parasite killing without untoward tissue inflammation.  Ref: 12379707 Infect Immun, 2002
- IL 22 and IL 10  [ **IL10** ]  require different ligand specific receptor chains IL 22R and IL 10R1  [ **IL10RA** ]  but share a second receptor chain IL 10R2 to initiate cellular responses.  Ref: 12513909 J Interferon Cytokine Res, 2002
- IL 10  [ **IL10** ]  signaling via IL 10E1 is dependent on tyrosine phosphorylation in the IL 10R  [ **IL10RA** ]  alpha chain in human primary prostate cancer cell lines.  Ref: 12802285 Oncogene, 2003
- This latter response to IL 10  [ **IL10** ]  was strictly dependent on the levels of expression of IL 10R1  [ **IL10RA** ] , which were in fact significantly increased by IL 4 in cultured neutrophils via a signaling pathway sensitive to the serine threonine kinase inhibitor H 7.  Ref: 12356680 Int Immunol, 2002
- Human IL 10  [ **IL10** ]  hIL 10 modulates critical immune and inflammatory responses by way of interactions with its high IL 10R1  [ **IL10RA** ]  and low affinity IL 10R2 cell surface receptors.  Ref: 12093920 Proc Natl Acad Sci U S A, 2002
- IL 10  [ **IL10** ]  is a dimeric cytokine that must engage its high affinity cell surface receptor, IL 10R1  [ **IL10RA** ] , to induce multiple cellular activities.  Ref: 12121653 Structure Camb , 2002
- Human cytomegalovirus exploits the IL 10  [ **IL10** ]  signaling pathway by expressing a functional viral IL 10  [ **IL10** ]  homolog cmvIL 10, which shares only 27% sequence identity with hIL 10 yet signals through IL 10R1  [ **IL10RA** ]  and IL 10R2.  Ref: 12093920 Proc Natl Acad Sci U S A, 2002
- In contrast to the high expression of receptors for IL 10  [ **IL10** ]  homologs in different tissues and cell lines, monocytes and NK, B, and T cells showed clear expression only of IL 10R1  [ **IL10RA** ] , IL 10R2, and IL 20R2.  Ref: 12023331 J Immunol, 2002
- We aimed to clone and sequence partial cDNA for rat IL 10R  [ **IL10RA** ]  and CRF2 4, determine their expression in activated rat HSC in vivo and in vitro, and examine the biological responsiveness of HSC to exogenous IL 10  [ **IL10** ] .  Ref: 12016123 Am J Physiol Gastrointest Liver Physiol, 2002
- IL 22 is an IL 10  [ **IL10** ]  homologue that binds to and signals via the class II cytokine receptor R heterodimer IL 22RA1 CFR2 4 IL 10R2, the latter chain being part of the IL 10R  [ **IL10RA** ]  complex.  Ref: 12407026 Int Immunol, 2002
- Cell proliferation and IL 10  [ **IL10** ]  production were compared in vitro, and tumour growth, percentages of necrotic areas, tumour cells positive for proliferating cell nuclear antigen PCNA, IL 10  [ **IL10** ]  receptor IL 10R  [ **IL10RA** ]  and major histocompatibility complex type I MHC I and II MHC II, as well as infiltration of macrophages, CD4 and CD8 lymphocytes and blood vessels were compared in vivo among IL 10  [ **IL10** ]  transfected and non transfected tumours.  Ref: 11872099 Immunology, 2002
- The production of IL 10  [ **IL10** ]  by THP 1 cells stimulated with L OspA was autoregulated by a negative feedback mechanism involving the IL 10  [ **IL10** ]  receptor IL 10R  [ **IL10RA** ] .  Ref: 11895951 Infect Immun, 2002
- The acute death of trypanosome infected mice treated with anti IL 10R  [ **IL10RA** ]  antibodies in vivo was associated with focal liver necrosis, with significantly increased plasma levels of IL 6, IL 10  [ **IL10** ] , IL 12p40 and IFN gamma and enhanced synthesis of IL 6, IL 12p40 and IFN gamma by spleen cell cultures.  Ref: 12594839 Eur J Immunol, 2003

**Non-interactive relationship** (e.g. studied together, co-existance, homology, etc.)

- Blocking of murine IL 10  [ **IL10** ]  receptor IL 10R  [ **IL10RA** ]  in vivo shortened the survival time of highly susceptible T. congolense infected BALB c mice.  Ref: 12594839 Eur J Immunol, 2003
- However, because anti IL 10R  [ **IL10RA** ]  also released IFN gamma independent effects, IL 10  [ **IL10** ]  appears to act more broadly and suppresses multiple antileishmanial mechanisms.  Ref: 12870129 J Infect Dis, 2003
- As judged by IL 10R  [ **IL10RA** ]  blockade, endogenous IL 10  [ **IL10** ]  primarily regulates killing in L. donovani infection by suppressing production of and responses to the Th1 cell type cytokines, IL 12, and IFN gamma.  Ref: 12870129 J Infect Dis, 2003
- The quaternary structures and the ability of IL 22 and IL 10  [ **IL10** ]  to engage soluble s IL 10R1  [ **IL10RA** ] , IL 22R, IL 10R2 receptor chains were analyzed using size exclusion chromatography and surface plasmon resonance techniques.  Ref: 12513909 J Interferon Cytokine Res, 2002
- In addition, exogenous IL 10  [ **IL10** ]  is highly potent in down regulating LPS induced IL 1beta and IL 10  [ **IL10** ]  mRNA, and, at a higher dose, IL 10R1  [ **IL10RA** ]  mRNA in untreated and LPS treated cultures, suggesting that IL 10  [ **IL10** ]  autoregulates its expression and inhibits that of IL 1beta at the transcriptional level.  Ref: 12405978 Eur J Neurosci, 2002
- IL 10R  [ **IL10RA** ]  expression did not correlate with plasma IL 10  [ **IL10** ]  levels or disease activity.  Ref: 12673894 Clin Exp Rheumatol< MedlineTA>Clin Exp Rheumatol, 2002
- OBJECTIVE We aimed to determine the expression of the interleukin 10  [ **IL10** ]  receptor IL10R  [ **IL10RA** ]  on circulating leukocytes in SLE and rheumatoid arthritis, and correlate this with plasma IL 10  [ **IL10** ]  levels and disease activity.  Ref: 12673894 Clin Exp Rheumatol< MedlineTA>Clin Exp Rheumatol, 2002
- Therefore, we have examined the expression of the anti inflammatory cytokines, interleukin 10  [ **IL10** ]  IL 10  [ **IL10** ]  and IL 4, and their specific receptors, IL 10R  [ **IL10RA** ]  and IL 4R, in postmortem human brain tissue obtained from MS patients.  Ref: 11921201 Glia, 2002
- During relapse, IL 10  [ **IL10** ] , IL 10R1  [ **IL10RA** ] , ICAM 1, VCAM 1 mRNA levels and neurological scores show positive correlations.  Ref: 12620647 J Neuroimmunol, 2003
- To develop a rationale for IL 10  [ **IL10** ]  inhibition as treatment in visceral infection, Th1 cytokine driven responses were characterized in Leishmania donovani infected BALB c mice in which IL 10  [ **IL10** ]  was absent or overexpressed or its receptor IL 10R  [ **IL10RA** ]  was blockaded.  Ref: 12379707 Infect Immun, 2002
- Immune suppression was mediated independently of IL 10  [ **IL10** ]  and TGF beta production, as neutralizing antibodies for IL 10  [ **IL10** ] , IL 10R  [ **IL10RA** ] , and TGF beta were unable to revert suppression, and IL 10  [ **IL10** ]  deficient CD4 T cells were able to mediate in vitro and in vivo suppression.  Ref: 12609834 Blood, 2003
- Furthermore, intravenous LPS upregulated expression of IL 10  [ **IL10** ]  receptor IL 10R1  [ **IL10RA** ]  mRNA in the liver.  Ref: 12010759 Am J Physiol Regul Integr Comp Physiol, 2002
- Interleukin 10  [ **IL10** ]  receptor IL 10R  [ **IL10RA** ]  expression in human, dental pulp, fibroblast cultures was investigated by using the reverse transcriptase polymerase chain reaction RT PCR technique.  Ref: 12540220 J Endod, 2003
- Structural analysis of the S138G variant revealed that the substitution of S138G may interfere with binding of IL 10  [ **IL10** ]  to IL 10R1  [ **IL10RA** ] .  Ref: 12759436 J Immunol, 2003
- In the present study we describe the expression of IL 10  [ **IL10** ]  and the IL 10  [ **IL10** ]  receptor IL 10R1  [ **IL10RA** ]  in primary cocultures of rat microglial and astroglial cells.  Ref: 12405978 Eur J Neurosci, 2002
- In established Leishmania donovani visceral infection in normal mice, anti interleukin IL 10  [ **IL10** ]  receptor IL 10R  [ **IL10RA** ]  monoclonal antibody MAb treatment induced intracellular parasite killing within liver macrophages.  Ref: 12870129 J Infect Dis, 2003
- IL 10  [ **IL10** ]  induced SOCS1 may directly inhibit IL 10  [ **IL10** ]  IFN gamma signaling, while inhibition of other proinflammatory cytokine responses may use additional IL 10R1  [ **IL10RA** ]  mediated mechanisms.  Ref: 12538698 J Immunol, 2003
- This shows concomitant induction of IL 10R  [ **IL10RA** ]  but not CRF2 4 to that of IL 10  [ **IL10** ]  by activated HSC in vitro and in vivo and associated acquisition of the responsiveness to IL 10  [ **IL10** ] , entailing complex effects on HSC.  Ref: 12016123 Am J Physiol Gastrointest Liver Physiol, 2002
- The significance of IL 10  [ **IL10** ]  and IL 10R  [ **IL10RA** ]  expression in renal cell carcinoma RCC has not been well characterized.  Ref: 11894117 Int J Oncol, 2002
- IL 10  [ **IL10** ]  s deactivating effects regulate outcome in experimental visceral leishmaniasis, and IL 10R  [ **IL10RA** ]  blockade represents a potential immuno and or immunochemotherapeutic approach in this infection.  Ref: 12379707 Infect Immun, 2002
- A single mutation Ile87Ala in the same CD loop region of the Epstein Barr virus IL 10  [ **IL10** ]  ebvIL 10 also reduces IL 10R1  [ **IL10RA** ]  binding affinity, suggesting that ebvIL 10 and 9D7Fab use similar allosteric mechanisms to modulate IL 10R1  [ **IL10RA** ]  affinity and biological activity.  Ref: 12121653 Structure Camb , 2002
- Collectively, our data emphasize the central role of IL 10R1  [ **IL10RA** ]  expression in regulating cell responsiveness to IL 10  [ **IL10** ] .  Ref: 12356680 Int Immunol, 2002
- To investigate the role of other loci in the susceptibility to RA, we have performed an analysis of several polymorphisms in genes of immune related function IL 10  [ **IL10** ]  108 1 92 promoter single nucleotide polymorphisms SNPs, IL 10G and IL 10R  [ **IL10RA** ]  microsatellites, IL 6 622 promoter SNP, FcgammaRIIIA Val Phe 158 polymorphism, IL 1 receptor antagonist VNTR, and the IKBL 738 T C mutation.  Ref: 12618859 Genes Immun, 2003
- In a rat model of chronic relapsing EAE, expression levels of IL 10  [ **IL10** ]  and its receptor IL 10R1  [ **IL10RA** ] , ICAM 1 and VCAM 1 mRNA in the spinal cord are markedly increased.  Ref: 12620647 J Neuroimmunol, 2003
- IL 10  [ **IL10** ]  induced SOCS is associated with the inhibition of IFN gamma signaling in various cell types, and this inhibition is independent of C terminal serine residues of the IL 10R  [ **IL10RA** ] , previously shown to be required for other anti inflammatory responses.  Ref: 12538698 J Immunol, 2003
- In IL 10R1  [ **IL10RA** ]  G330R expressing monocytes, the inhibitory effect of IL 10  [ **IL10** ]  on TNF alpha production was diminished, indicating that this variant may be a loss of function allele.  Ref: 12759436 J Immunol, 2003
- We studied three SNPs  **IL10**  108 24, and 597 and two microsatellites IL10R  [ **IL10RA** ]  and IL10G lying within the promoter region of  **IL10**  for association with susceptibility to and clinical manifestations of ankylosing spondylitis AS, a common form of spondyloarthritis.  Ref: 12595905 Genes Immun, 2003
